# Critical discourse analysis of social media advertisements for GLP-1 receptor agonist weight loss drugs: implications for public perceptions and health communication

**DOI:** 10.1186/s12889-025-24197-8

**Published:** 2025-09-01

**Authors:** J. Rad, G. J. Melendez-Torres

**Affiliations:** Faculty of Health and Life Sciences, University of Exeter, Exeter, UK

**Keywords:** Critical discourse analysis, Social media advertising, GLP-1 receptor agonist, Weight loss drugs, Health communication, Telehealth

## Abstract

This study conducts a critical discourse analysis (CDA) of social media advertisements (ads) for Glucagon-like peptide-1 receptor agonist (GLP-1 RAs) weight loss drugs to examine their linguistic features, discursive practices, and social implications, aiming to explore how advertising strategies may shape public perceptions and consumer considerations. Data collection focused on Facebook and Instagram, targeting ads related to FDA-approved GLP-1 RAs weight loss drugs from February 2023 to February 2024. Ads were collected using specific keyword searches. Inclusion criteria focused on ads explicitly related to the three GLP-1 RAs weight loss drugs, while exclusion criteria removed irrelevant, non-English, duplicate, or incomprehensible ads. Thematic analysis was conducted to identify recurring themes. Fairclough’s CDA model was used to analyse the 90 ads. Thematic analysis identified four key themes including “Health and Safety”, “Consultation and Support”, “Ease and Affordability”, and “Emotional and Psychological Impact”. These themes highlight the emphasis on medical credibility, accessibility through telehealth, affordability, and emotional benefits. The ads strategically use medical authority and emotional appeals, seemingly to build trust and persuade consumers. The focus on telehealth aligns with the growing demand for remote healthcare solutions, while the emphasis on affordability and ease of use addresses common barriers to accessing weight loss treatments. This research provides new insights into the discursive strategies of pharmaceutical advertising, distinguishing it as a unique contribution to the field. The study underlines the need for balanced health communication that accurately represents the benefits and risks of weight loss drugs. It recommends stricter regulatory guidelines to ensure transparency and prevent exaggerated claims. Additionally, public health campaigns should educate consumers on comprehensive weight management, integrating medication with lifestyle changes. Moreover, stakeholders can develop better health communication strategies and regulatory policies to enhance public health outcomes by understanding the narratives in these ads.

**Supplementary Information:**

The online version contains supplementary material available at 10.1186/s12889-025-24197-8.

## Introduction

The prevalence of weight-related health issues has become a significant public health concern in recent years, with obesity and associated conditions such as cancer, diabetes, hypertension, stroke, cardiovascular disease, and psychological problems affecting a growing number of individuals worldwide [1–5].

Despite various efforts to address these issues, obesity rates continue to rise, driving the need for effective pharmaceutical interventions. One promising class of drugs that has gained attention for their efficacy in promoting weight loss and managing diabetes is Glucagon-like peptide 1 receptor agonists (GLP-1 RAs) [6–10]. These drugs mimic the action of the glucagon-like peptide-1 hormone [11], which regulates appetite and insulin secretion, making them valuable in the fight against obesity and its related conditions.

The financial aspect of GLP-1 RAs is substantial and growing. Sales have surged in recent years, reflecting their increasing acceptance and demand. In early 2023, the global market for anti-obesity medications was $6 billion, with projections suggesting it could reach $100 billion by 2030 [12]. The market for drugs like Ozempic and Wegovy is expected to hit $100 billion by 2035 due to increased patient awareness [13]. According to a Reuters report on February 6, 2024, Eli Lilly anticipates a strong 2024 due to high demand for its new weight-loss drug, Zepbound, with initial sales reaching $175.8 million [14].

As the impact of these conditions continues to expand, so does the demand for effective weight loss solutions. In this context, pharmaceutical advertisements (ads) are crucial in shaping public perceptions of health, beauty, and weight management [15]. These ads influence consumer behaviour and contribute to the broader discourse surrounding weight loss and health interventions.

With the increasing popularity of social media platforms, ads for weight loss drugs are reaching more extensive and diverse audiences. This broad reach has led to a flood of messages that can shape consumer attitudes and expectations regarding weight management. However, the strategic use of language, imagery, and promotional tactics in these ads raises essential questions about the commercial determinants of health (CDOH). The CDOH framework examines how commercial interests shape health outcomes and health inequities, providing a critical lens to understand the impact of corporate practices on public health.

This phenomenon can also be understood within the broader framework of the pharmaceuticalization of society, a process in which health and social problems are increasingly addressed through pharmaceutical solutions [16]. The aggressive promotion of GLP-1 RA weight loss drugs reflects this trend, where complex issues such as obesity and body image dissatisfaction are medicalized and positioned for pharmaceutical intervention. This framing influences both individual health behaviors and broader public health discourses.

Recent research underscores the significant role of emotional appeals and source credibility in shaping public responses to health advertising on social media platforms. Odunfa in [17] demonstrated that fear and hope appeals in health messages can significantly influence college students’ attitudes and intentions regarding health behaviors. Similarly, Hosseini and Staab [18] found that emotionally framed health claims, whether true or false, are more likely to be shared on social media, highlighting the persuasive power of emotional content. In terms of credibility, the National Academy of Medicine [19] proposed a framework for identifying trustworthy health information sources on social media, emphasizing transparency and accountability. Kuutila et al. [20] further revealed that adults’ evaluations of health-related social media posts are influenced by source expertise and prior beliefs, affecting the perceived credibility of the information. Integrating these insights provides a deeper understanding of how GLP-1 RA advertisements on social media may impact public perceptions and health-related decision-making.

Critical discourse analysis (CDA) plays a crucial role in exploring these issues, offering a robust framework to investigate how these ads construct and convey their messages. CDA focuses on the power relations and ideologies that underlie discourse, allowing us to understand how language can exert power, maintain social hierarchies, and perpetuate dominant ideologies [21–27]. By examining these ads, CDA reveals the implicit messages and rhetorical strategies that drive consumer behaviour, often in ways that serve commercial interests rather than public health. This study aims to uncover the marketing strategies and commercial interests behind these ads using CDA, providing insight into weight-related health issues.

## Methodology

### Data collection

Data collection focused on Facebook and Instagram, two major platforms known for their extensive digital advertising reach and diverse user demographics. Keyword searches were systematically conducted on a monthly basis throughout the one-year period (February 24, 2023-February 24, 2024) to capture a comprehensive and dynamic sample of advertisements and ensure timely inclusion of newly launched campaigns.

Keyword and hashtag selection was guided by relevance, frequency of use, and popularity within social media contexts related to weight loss and pharmaceutical marketing. Initial exploratory searches identified frequently used keywords and hashtags relevant to GLP-1 receptor agonist drugs. After this exploratory stage, a refined set of keywords and hashtags was finalized to capture the most relevant advertisements comprehensively. The final set included generic drug names (e.g., liraglutide, semaglutide, tirzepatide), brand names (Ozempic, Wegovy, Zepbound), and hashtags commonly associated with weight-loss discourse (e.g., *#weightloss*, *#GLP1*, *#OzempicJourney*, *#WeightLossJourney*).

For Facebook, data collection was conducted exclusively through the *Facebook Ad Library*, a publicly accessible database that archives only paid advertisements, ensuring that the collected content represented promotional materials with commercial intent. On Instagram, we identified and collected posts explicitly marked as *“*Paid Partnership*”* or labeled as sponsored content, following the platform’s advertising disclosure guidelines. Posts without clear commercial intent or those not directly associated with GLP-1 RA drugs were excluded during the screening process.

All collected advertisements were securely stored by saving visual content (screenshots of advertisements) and archiving the corresponding textual content in a dedicated research database for further analysis. To maintain data integrity and relevance, we applied strict inclusion criteria focusing solely on commercial content explicitly designed to promote GLP-1 RA weight loss products. User-generated content, non-commercial posts, and posts without identifiable promotional intent were systematically excluded from the dataset during the initial review phase. Specific inclusion and exclusion criteria are summarized in Table 1. During the screening process, irrelevant posts were systematically excluded based on the following criteria:

1. Lack of direct reference to GLP-1 receptor agonists or their commercial brand names (e.g., Ozempic, Wegovy, Zepbound).
2. Absence of clear commercial or promotional intent, identified by the lack of “Paid Partnership” labels or other indicators of advertising content.
3. Content not in English or posts with incomprehensible text or visuals.
4. Duplicate advertisements or posts that did not meet the inclusion criteria outlined in Table 1.

**Table 1:** Inclusion and exclusion criteria for advertisement selection

Inclusion criteria	Exclusion criteria
Advertisements with content related to GLP-1 RAs weight loss drugs	Non-English advertisements
Advertisements explicitly related to GLP-1 RAs weight loss drugs	Advertisements irrelevant to GLP-1 RAs weight loss drugs
Ads providing detailed visuals, textual content, and claims	Duplicate advertisements/Incomprehensible ads

#### Data collection outcomes

**Fig. 1:**
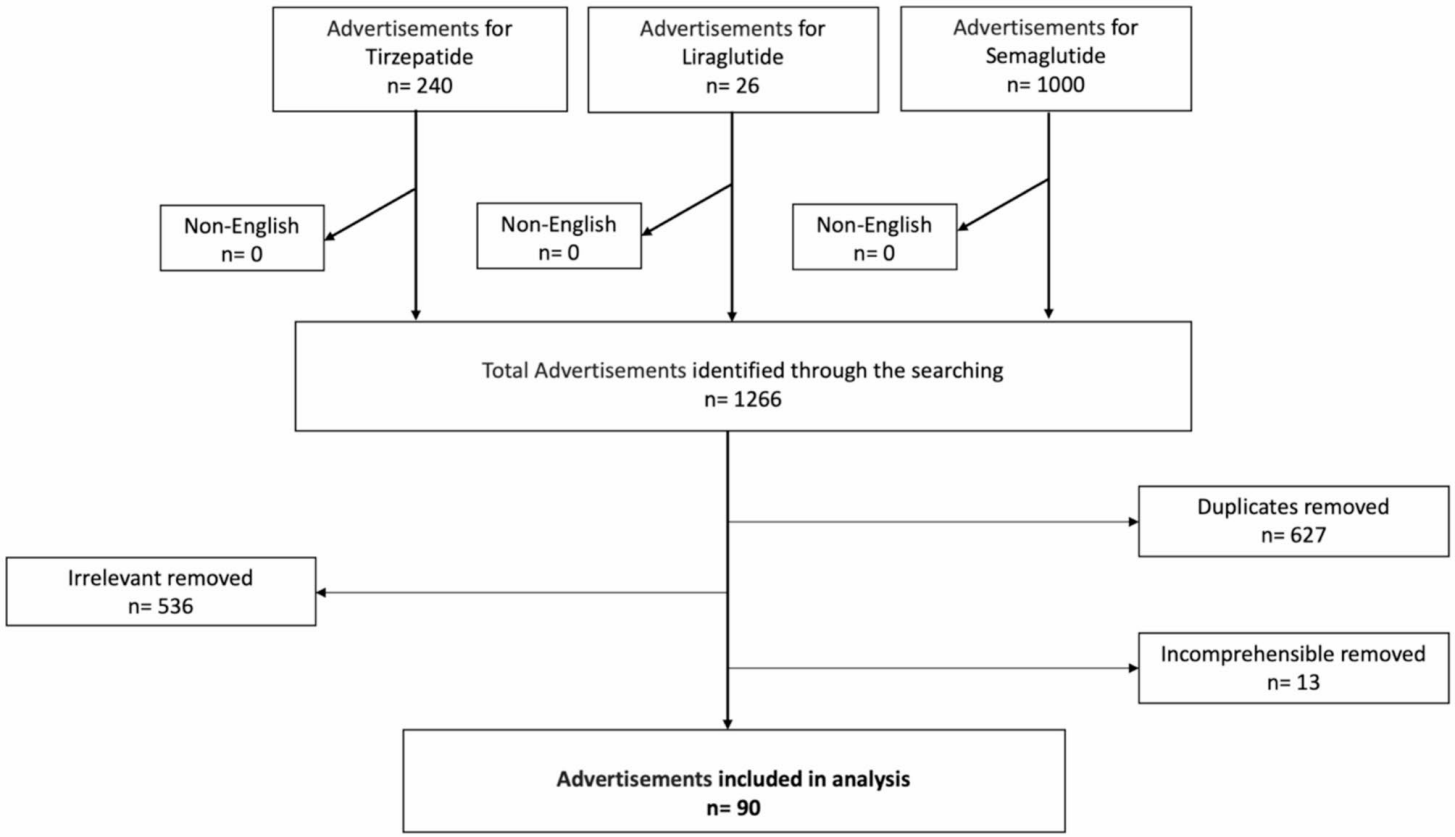
The advertisement data collection and selection process

The selection process for ads is detailed in the flow diagram (Fig. 1). An initial dataset of 1,266 advertisements was gathered from Facebook and Instagram based on the specified keywords and hashtags. A rigorous screening process was conducted to ensure analytic relevance and clarity, applying clearly defined inclusion and exclusion criteria summarized in Table 1.

Inclusion criteria mandated advertisements explicitly promoting GLP-1 receptor agonist drugs for weight loss, clear commercial intent, and English-language content.

Exclusion criteria eliminated advertisements lacking explicit commercial intent, duplicates, advertisements unrelated directly to GLP-1 drugs, those without identifiable promotional objectives, non-English ads, and advertisements with unclear or incomprehensible visual or textual content. This strict screening protocol ensured coherence in our analytic sample, resulting in a refined dataset of 90 unique advertisements most representative of the commercial discourse surrounding GLP-1 RA weight-loss medications.

### Data analysis

Thematic analysis was performed by identifying initial codes and categorising them into themes. Following Saldaña’s [28] approach, an NVivo codebook was inductively developed. This involved extracting recurring words, phrases, and concepts from the ads to identify initial codes, grouping these codes into broader themes based on their similarities, and reviewing and refining the themes to ensure they accurately represented the data. Additionally, CDA was employed, utilizing Wodak and Meyer’s framework (24). The coding process began with an open, inductive reading of the data to identify recurring patterns and discursive strategies. Initial codes were generated independently by two researchers and then discussed collaboratively to develop a shared understanding of thematic categories. A codebook was created during this process, containing clear definitions and representative examples for each theme. This codebook was refined through iterative cycles of coding and discussion using NVivo 12 software. As new subthemes emerged, they were incorporated into the codebook, and overlapping codes were consolidated to maintain coherence across the dataset.

To ensure methodological rigor, inter-coder reliability was systematically assessed. Two independent coders performed thematic and visual coding on a randomly selected subset comprising approximately 20% of the total dataset. Inter-coder agreement was quantified using Cohen’s Kappa coefficient [29], resulting in a robust reliability score of 0.85, indicating strong agreement. Coding discrepancies were collaboratively discussed and resolved through consensus meetings, resulting in a clearly defined and consistently applied thematic and visual codebook.

Thematic saturation was systematically assessed through iterative cycles of coding. Saturation was deemed achieved when subsequent coding of advertisements yielded no novel themes, sub-themes, or additional analytical insights. Coders independently verified the point of saturation, confirmed via consensus meetings, thus ensuring comprehensiveness and depth of thematic representation across the analyzed dataset.

To ensure conceptual clarity, we distinguished between the two approaches: thematic analysis was used to identify what topics or recurring content appeared in the advertisements, while CDA was applied to examine how such content was linguistically and visually constructed to convey meaning, reinforce ideologies, and normalize certain values. This distinction between content-focused analysis (themes) and discourse-focused analysis (discourses) addresses the important methodological feedback raised during internal and peer review.

Building on this, we applied Fairclough’s three-dimensional framework for CDA, which involves three interconnected levels of analysis:

- Textual analysis: This focused on examining the linguistic characteristics of the advertisements, including frequent use of promotional language (e.g., “*clinically proven*,” “*safe and effective*”), the application of modal verbs (such as *can* and *will* to convey certainty), and the use of imperative sentences (e.g., “*Get started today*,” “*Order now and gain 7% discount*”) to prompt direct action.
- Discursive practice: This level analyzed how the advertisements are produced and consumed in the context of social media platforms. It considered how commercial advertising strategies on Facebook and Instagram integrate with emerging telehealth services and digital health marketing trends to influence consumer behaviors.
- Social practice: This level explored how the advertisements reinforce broader societal discourses related to ideal body image, health responsibility, and the normalization of pharmacological weight loss solutions. It also examined how the ads appeal to societal expectations regarding slimness, confidence, and personal transformation, contributing to the perpetuation of body image ideals.

In summary, thematic analysis enabled us to categorise recurring content across the dataset, while CDA allowed us to critically interrogate the rhetorical construction of that content and its social implications. This dual approach ensured that we captured both the surface-level themes and the deeper discursive mechanisms at play.

Insights from the CDA were integrated into the results and discussion sections to provide a deeper understanding of the social implications of the advertising strategies and the persuasive power of language and visual representation in shaping public perceptions.

### Visual analysis procedures

Visual analysis involved systematic coding of visual elements within the ads. Two coders independently analyzed key visual aspects such as imagery (e.g., depictions of individuals before/after weight loss), color usage (e.g., colors symbolizing health, urgency, or positivity), textual placement (e.g., headline prominence, call-to-action buttons), and emotional appeal (e.g., confidence, hope). Analysis followed established visual rhetoric methodologies [30]. Each visual element was recorded using NVivo qualitative software, and any discrepancies were resolved by consensus discussion.

### Ethical considerations

Our research ethics were guided by the principles outlined by Townsend and Wallace [31], and approval was obtained from the University of Exeter Medical School Research Ethics Committee. Only publicly available ads were collected, ensuring no personal data from social media users was included. Any identifiable information within the ads was anonymised to protect privacy.

## Results

### Key themes overview

Our analysis identified four key themes in the ads for GLP-1 RAs weight loss drugs on Facebook and Instagram. These themes include “Health and Safety” (“HS”), “Consultation and Support” (“CS”), “Ease and Affordability” (“EA”), and “Emotional and Psychological Impact” (“EPI”) (Table 2). Although this study is grounded in qualitative thematic analysis, we report approximate proportions of theme occurrences to illustrate the prominence of certain marketing strategies across the dataset. These figures are presented solely for descriptive purposes to support transparency and provide context and are not intended to imply statistical generalizability or causation.

**Table 2:** Themes, sub-themes, quotes examples, frequencies and weighted presence of themes and sub-themes in advertisements along with emotion/evocation and marketing strategy

Themes	Sub-themes	Quotes examples	Reference frequency^a^	Weighted presence^b^	Emotion/Evocation	Marketing strategy
Health and Safety	medical	*“Transform your weight and reclaim your health with the ultimate* ***medical*** *weight loss solution. For just S199/month unlock a new you!”* *“Semaglutide Assisted* ***Medical*** *Weight Management - A Game changer”* *“Semaglutide Weight Loss for Missouri Residents! We are taking on 15 Patients this month for our* ***Medical*** *Weight Loss Program. This program is designed for the person who has struggled with diets and exercises and the constant Yo-Yo.”* *“Semaglutide-Driven* ***Medical*** *Weight Management: The Future Is Now.”*	26	18.3%	Trust	Leveraging medical authority
	health	*“Transform your weight and reclaim your* ***health*** *with the ultimate medical weight loss solution.”* *“Exciting News for* ***Health*** *and Wellness Enthusiasts!”* *“Book a call with Victory Men’s Health today and get one of the best teams out there that focus strictly on men’s* ***health***.*”*	18	12.67%	Hope	Emphasizing health benefits
	safe	*“Semaglutide is a weekly injection clinically proven for* ***safe*** *and effective weight loss.”* *“****Safe*** *and effective once weekly injection*,* at home.”* *“Wegovy - Fast and* ***safe*** *way to lose weight. “*	5	3.52%	Security	Highlighting safety assurances
	effective	*“Semaglutide is a weekly injection clinically proven for safe and* ***effective*** *weight loss.”* *“For a limited time*,* get 50% off Tirzepatide*,* the newest*,* most highly* ***effective*** *weight loss peptides!”* *“Completely Safe and Remarkably* ***Effective****”*	13	9.15%	Confidence	Demonstrating efficacy
Consultation and Support	consultation	*“Get started today with a free* ***consultation***.*”* *“Virtual physician* ***consultation*** *and lab work included.”* *“Then we do a virtual* ***consultation*** *where we can come up with your individualized plan for weight loss.”*	22	15.49%	Reassurance	Offering professional support
	online	*“Prescription products require an* ***online*** *consultation with a doctor.”* *“All* ***online****! Medication options in-stock”* *“Experience the Power of Semaglutide: Real Results*,* Real Convenience. Prescribed* ***Online***, *Delivered to Your Doorstep.”*	18	12.67%	Convenience	Promoting accessibility
	support	“*We’re here to monitor your progress and* ***support*** *you every step of the way.”* *“Lose weight efficiently and safely with the* ***support*** *of Juna’s board-certified doctors*,* who offer tailored solutions one shot at a time”* *“Our team of clinicians will create a personalized plan just for you*,* providing the best medication*,* and offer continuous* ***support*** *to ensure your success.”*	8	5.63%	Comfort	Providing ongoing assistance
Ease and Affordability	easy	*“Discover semaglutide for* ***easy*** *and erective assistance on your journey to long-term weight management”* *“It’s quick*, ***easy***, *and could be the first step toward the compensation you deserve. Act now!”* *“How to Start…Easy as 1*,* 2*,* 3!”*	6	4.22%	Simplicity	Simplifying the process
	affordable	*“****Affordable*** *and effective*,* start your journey today!”* *“Slim down with our* ***affordable*** *weight loss injections!”* *“Have you been wondering where to get GLP-1s at an* ***affordable*** *price? Look no further.”*	5	3.52%	Value	Highlighting affordability
	discount	*“we got pen available in stock. order now and gain 7%* ***discount***. *Let’s enjoy healthy life together and be happy and healthy.”*	1	0.7%	Urgency	Offering special promotions
Emotional and Psychological Impact	confidence	“*Experience significant weight reduction*,* fewer serious side effects*,* and a new level of* ***confidence***.” “*Our satisfied customers report reduced joint pain*,* enhanced energy levels*,* improved sexual health*,* increased* ***confidence***, *and optimized cardiac health.*” “***Confidence*** *Boost: Shedding pounds and feeling fantastic is the ultimate reward. Embrace the new you!*”	4	2.81%	Confidence	Building self-esteem
	transformation	*“Attention Harlingen Ladies*,* it’s time to kickstart your* ***transformation****!”* *“Join thousands who have found success with Mounjaro– start your* ***transformation*** *today!”* *“Start an empowering* ***transformation*** *towards a renewed you!”*	4	2.81%	Transformation	Emphasizing life-changing results
	cravings	“*Suppress appetite and* ***cravings***” “*Reduces appetite*,* improves control of eating and reduces food* ***cravings***.” “*Feel fuller faster*,* curb your appetite*,* and say goodbye to* ***cravings***.*”*	12	8.45%	Control	Addressing hunger control
Note: ^a^The proportion of each sub-theme’s mentions relative to the total number of themes mentions, expressed as a percentage						
^b^The proportion of each sub-theme’s mentions relative to the total number of themes mentions, expressed as a percentage						

To support the thematic analysis, anonymised sample images of selected advertisements have been included as supplementary material (Supplementary Figure S1), illustrating key visual and textual strategies corresponding to the identified themes.

#### Health and safety

The most prominent theme in the ads was “Health and Safety” (“HS”), with frequent references to medical credibility and efficacy. The terms “*medical*”, “*health*”, “*safe*”, and “*effective*” were recurrently used (Table 2). These ads emphasized the drugs’ FDA approval and clinical backing to build trust and reassure consumers about the safety and reliability of these weight loss solutions. This theme was the most frequently observed across the advertisements, reflecting the emphasis placed on medical credibility and efficacy. This strategic emphasis suggests that ads aim to leverage medical authority to enhance the perceived legitimacy of their products. By highlighting FDA approval and clinical evidence, the ads seek to establish a foundation of trust, making the products appear more credible and trustworthy to potential consumers. The CDA reveals that these ads are crafted to construct persuasive narratives that resonate with consumer desires for safe, professional, and effective weight loss solutions. The frequent use of authoritative terms underscores a deliberate effort to leverage medical authority. For instance, phrases like “*Semaglutide-Driven Medical Weight Management: The Future Is Now*” and “*Semaglutide is a weekly injection clinically proven for safe and effective weight loss*” are designed to reinforce the medical legitimacy of these treatments.

#### Consultation and support

Another significant theme was “Consultation and Support” (“CS”), highlighted by the frequent mention of “*consultation*” and “*online*”. Many ads promoted the availability of professional consultations, often through online platforms, underscoring the accessibility and convenience of obtaining medical advice. This theme was frequently emphasized, particularly highlighting accessibility and the growing prominence of telehealth services. This theme reflects a broader trend towards telehealth services, which have gained prominence, especially during and after the COVID-19 pandemic. By offering easy access to consultations, these ads address potential barriers to seeking professional help for weight management. The focus on online consultations and telehealth services aligns with contemporary healthcare trends, emphasising accessibility and convenience. This resonates with consumers seeking safe and effective weight loss solutions that fit into their busy lifestyles, particularly in the wake of the COVID-19 pandemic.

#### Ease and affordability

The theme of “Ease and Affordability” (“EA”) was also prevalent. Ads frequently used terms like “*easy*”, and “*affordable*,” and occasionally mentioned discounts. This theme was less frequently highlighted but played an important role in addressing cost-related concerns among consumers. This theme highlights the efforts to make weight loss treatments appear straightforward and financially accessible. The mention of special pricing and discounts indicates a marketing strategy aimed at attracting cost-conscious consumers and those who might be hesitant to spend on long-term weight loss programs. The ads aim to lower the perceived barriers to entry, encouraging more consumers to consider these weight loss solutions by presenting the treatments as easy to follow and financially accessible. Consumers are significantly influenced by price incentives in health-related purchases, indicating that financial accessibility is a crucial factor in healthcare decisions.

#### Emotional and psychological impact

The “Emotional and Psychological Impact” (“EPI”) theme was characterized by references to “*confidence*”, “*transformation*”, and managing “*cravings*”. This theme was prominently used to appeal to consumers’ emotional motivations for weight loss and self-improvement. Ads often promise significant emotional and psychological benefits, suggesting that using these drugs would lead to enhanced self-esteem and a transformative journey. This theme taps into the emotional drivers behind weight loss, acknowledging the psychological aspects of weight management and the desire for personal transformation. By highlighting the potential for increased confidence and significant personal change, the ads appeal to the deeper emotional motivations of consumers, encouraging them to view the weight loss journey as a path to a better, more confident self.

The promotion of GLP-1 RA weight-loss drugs frequently intersects with broader socio-cultural narratives that equate slimness with success, attractiveness, and social acceptance. These advertisements often position weight loss not solely as a health goal but as a means to achieve improved self-confidence, desirability, and social mobility. Such messaging reinforces long-standing societal beauty ideals and may contribute to body dissatisfaction, particularly among vulnerable populations. Future research could further explore how pharmaceutical advertising participates in the construction and perpetuation of these cultural body image standards.

Emotional appeals play a significant role in these ads, tapping into deeper psychological drivers such as confidence, transformation, and control. By promising significant emotional and psychological benefits, the ads create a compelling appeal that addresses both rational and emotional aspects of consumer decision-making. Statements like “*Confidence Boost: Shedding pounds and feeling fantastic is the ultimate reward. Embrace the new you!*” and “*Start an empowering transformation towards a renewed you!*” are crafted to evoke feelings of self-improvement and emotional well-being.

These themes collectively illustrate a comprehensive strategy employed by advertisers in their social media ads. By addressing both the rational and emotional needs of consumers, these ads aim to create a compelling narrative that not only promotes the efficacy and safety of the products but also resonates with the personal aspirations and practical concerns of potential users.

The ads employ a range of sophisticated linguistic and visual techniques to enhance their persuasive impact. Imperative sentences, such as “*Get started today with a free consultation*” and “*order now and gain 7% discount*” create a sense of urgency and direct action. These techniques include the use of direct calls to action, a standard practice in marketing strategies across various industries. While such persuasive elements are not unique to GLP-1 RA advertisements, their application within the pharmaceutical weight-loss market raises important considerations regarding the medicalization of lifestyle issues and the framing of body image ideals. Modal verbs like “*can*” and “*will*” convey certainty and assurance, while promotional language highlights the products’ unique selling points. Visually, the ads often feature images of healthy, happy individuals, reinforcing the desired outcomes of using weight loss drugs. These techniques are consistent with effective advertising practices that aim to engage and persuade the target audience through clear and compelling messages.

Moreover, addressing psychological needs is critical to the ads’ strategy. By highlighting the emotional and psychological benefits of the weight loss treatments, the ads aim to connect with consumers on a deeper level. The focus on increased confidence, personal transformation, and control over cravings addresses the underlying psychological motivations for weight loss. This approach not only enhances the appeal of the products but also aligns with consumers’ broader goals of self-improvement and emotional well-being. Such strategies are effective in motivating consumers but also raise ethical concerns about exploiting vulnerabilities related to body image and self-esteem.

The CDA reveals that these ads do not operate in a vacuum; they are deeply embedded within broader socio-cultural norms and pressures around body image and health. By reinforcing societal expectations of slimness and attractiveness, the ads tap into prevailing cultural narratives that equate weight loss with improved self-worth and social acceptance. This socio-cultural reinforcement plays a crucial role in shaping consumer perceptions and behaviours, as individuals internalise these messages and align their health goals with societal standards.

Finally, the analysis highlights the tension between commercial interests and public health discourse. While the ads effectively build trust and credibility through medical authority and emotional appeals, there is a notable lack of balanced information regarding potential side effects and risks associated with GLP-1 RAs drugs. This omission could lead to unrealistic expectations and undermine consumer trust if adverse effects are experienced without prior warning. The findings underscore the need for ethical advertising practices that prioritize transparency and consumer well-being over commercial gains. Ensuring balanced health communication is essential to maintaining consumer trust and promoting informed decision-making (Table 3).

**Table 3:** Summary of critical discourse analysis insights. It outlines the key focus areas and key insights derived from the CDA of social media advertisements for GLP-1 receptor agonist weight loss drugs

Key focus areas	Key insights
Strategic construction of narratives	Advertisements use authoritative terms to build trust and credibility by emphasizing medical authority.
Emotional appeals	Advertisements tap into psychological drivers such as confidence, transformation, and control to create compelling appeals.
Linguistic and visual techniques	Use of imperative sentences, modal verbs, and promotional language enhances persuasive impact.
Addressing psychological needs	Emphasizing emotional and psychological benefits connects with deeper consumer motivations.
Consumption context and resonance	Focus on online consultations and telehealth services aligns with contemporary healthcare trends.
Socio-Cultural reinforcement	Advertisements reinforce societal norms around body image and health, shaping consumer perceptions and behaviors.
Commercial interests and public health discourse	The tension between commercial interests and public health is evident, highlighting the need for balanced information and ethical advertising practices.

### Visual rhetoric

Visual rhetoric in ads consistently featured positive imagery (smiling individuals, before-after comparisons) to evoke emotional responses such as hope, transformation, and confidence. Color schemes emphasizing health (greens, whites) and urgency (reds, yellows) were strategically employed to reinforce textual messages and create persuasive appeals.

### Emotion/evocation

**Fig. 2:**
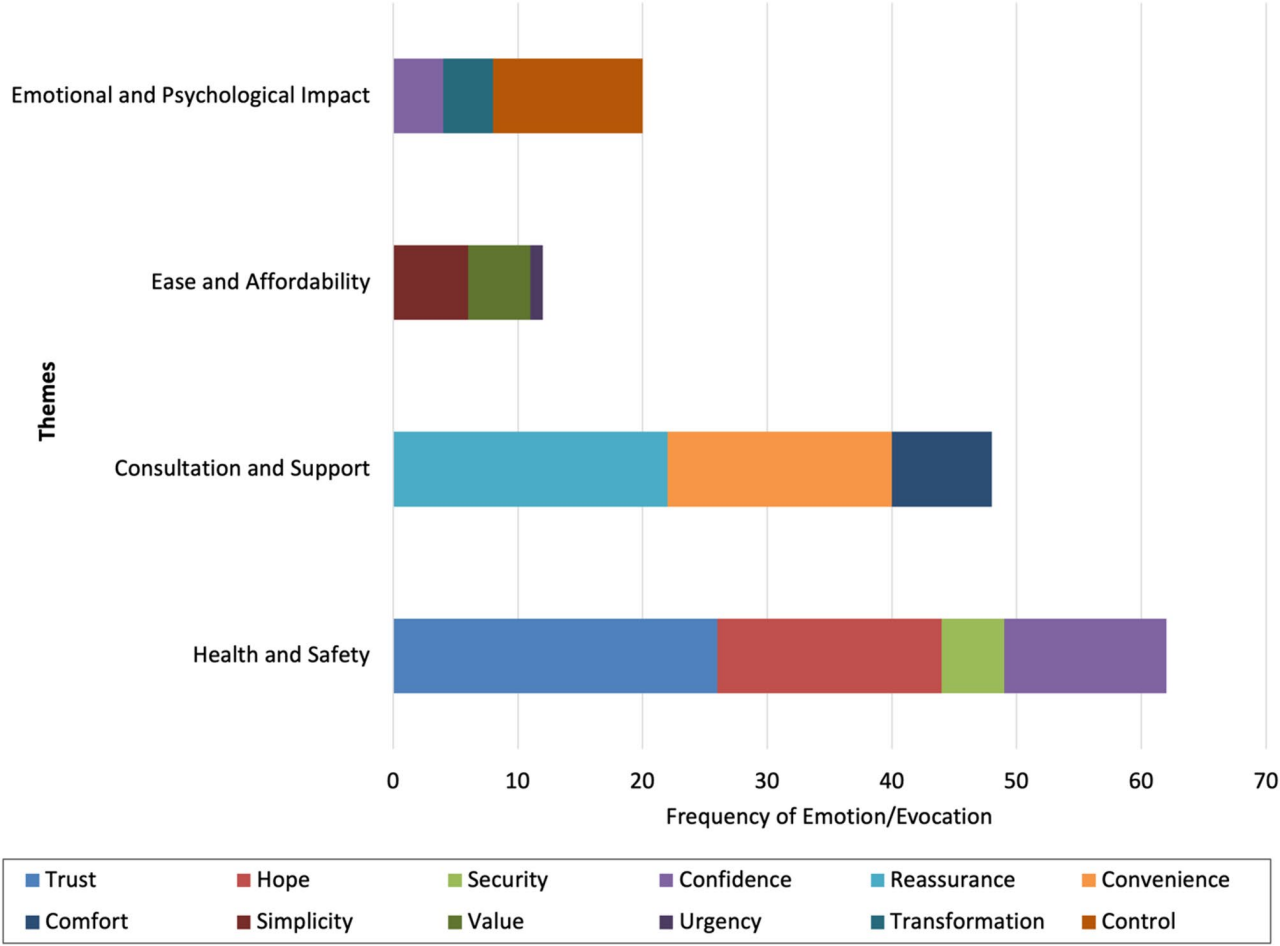
The contribution of different emotions to each theme in the advertisements. It highlights the dominant emotions evoked by each theme, providing insights into the strategic emotional appeals used in the marketing of GLP-1 receptor agonist weight loss drugs

Each theme in GLP-1 RAs weight loss drug ads evokes specific emotions, influencing consumer perceptions and behaviors. The “HS” theme evokes trust by leveraging medical authority, building credibility, and reassuring consumers about safety and efficacy. Within this theme, the “*health*” sub-theme evokes hope by fostering optimism about improved health outcomes. The “*safe*” sub-theme evokes security by alleviating safety concerns, while the “*effective*” sub-theme instils confidence by demonstrating the efficacy of the treatments. The “CS” theme evokes reassurance by emphasizing professional support, offering comfort through expert guidance. Within this theme, the “*consultation*” sub-theme highlights the availability of professional advice, evoking reassurance. The “*online*” sub-theme underscores convenience, emphasizing ease of access to consultations, while the “*support*” sub-theme evokes comfort by highlighting ongoing assistance throughout the weight loss journey. The “EA” theme evokes simplicity and value, emphasizing an uncomplicated process and financial accessibility. The “*easy*” sub-theme underscores simplicity, making the weight loss treatments appear straightforward. The “*affordable*” sub-theme highlights the value, appealing to budget-conscious consumers, and the “*discount*” sub-theme evokes urgency, encouraging immediate action with special promotions. The “EPI” theme addresses deeper psychological needs, evoking confidence, transformation, and control. The “*confidence*” sub-theme fosters self-esteem by promising significant personal improvement. The “*transformation*” sub-theme emphasizes life-changing results, appealing to consumers’ desire for a profound change. The “*cravings*” sub-theme evokes control by suggesting that the treatments can help consumers manage their eating habits effectively (Table 2; Fig. 2).

### Marketing strategy

The marketing strategies in social media ads for GLP-1 RAs weight loss drugs target consumer emotions and needs, positioning the products as credible and effective. These strategies align with identified themes and use various techniques to enhance appeal and drive engagement (Tables 2 and 4).

A significant strategy involves leveraging medical authority to build trust and credibility. Ads frequently highlight the medical aspects of the drugs. For instance, phrases like “*Semaglutide-Driven Medical Weight Management: The Future Is Now*” and “*Safe and effective once weekly injection*,* at home*” are used to underscore the medical credibility and safety of the treatments. This approach is designed to evoke trust and confidence in potential users, positioning the drugs as reliable and scientifically backed solutions.

The strategy of offering professional support is evident in the emphasis on consultations and ongoing assistance. Ads promote the availability of professional consultations and online support, with statements like “*Virtual physician consultation and lab work included*” and “*Our team of clinicians will create a personalized plan just for you*,* providing the best medication*,* and offer continuous support to ensure your success*”. This strategy aims to reassure consumers by emphasizing the professional and personalized nature of the support provided, thereby enhancing the perceived value of the product.

To appeal to cost-conscious consumers, the ads highlight the ease of use and affordability of the weight loss treatments. Phrases such as “*How to Start…Easy as 1*,* 2*,* 3!*” and “*Affordable and effective*,* start your journey today!*” are used to communicate simplicity and value. By emphasizing special offers and discounts, such as “*order now and gain 7% discount*” the ads create a sense of urgency and make the treatments seem more accessible and financially feasible.

The emotional and psychological impact is a crucial component of the marketing strategy. Ads tap into deeper psychological drivers by promising significant emotional and psychological benefits. For example, messages like “*Confidence Boost: Shedding pounds and feeling fantastic is the ultimate reward. Embrace the new you!*” and “*Join thousands who have found success with Mounjaro– start your transformation today!*” are designed to evoke feelings of confidence, transformation, and control. This strategy appeals to consumers’ desires for personal improvement and emotional well-being, making the treatments more attractive.

**Table 4:** Marketing strategies for GLP-1 receptor agonist weight loss drugs

Theme	Marketing Strategy Description	Example Quotes
Health and Safety	Highlighting medical credibility and safety to build trust and confidence.	*“Semaglutide-Driven Medical Weight Management: The Future Is Now.”* *“Safe and effective once weekly injection*,* at home.”*
Consultation and Support	Promoting the availability of professional consultations and ongoing support to reassure consumers of personalized and professional assistance.	*“Virtual physician consultation and lab work included.”* *“Our team of clinicians will create a personalized plan just for you*,* providing the best medication*,* and offer continuous support to ensure your success.”*
Ease and Affordability	Emphasizing the ease of use and affordability of treatments. Special offers and discounts to create a sense of urgency and financial accessibility.	*“How to Start…Easy as 1*,* 2*,* 3!”* *“Affordable and effective*,* start your journey today!”* *“order now and gain 7% discount.”*
Emotional and Psychological Impact	Tapping into psychological drivers by promising emotional benefits like confidence, transformation, and control to appeal to desires for personal improvement and well-being.	“*Confidence Boost: Shedding pounds and feeling fantastic is the ultimate reward. Embrace the new you!*” *“Join thousands who have found success with Mounjaro– start your transformation today!”*

## Discussion

This study examines social media ads for GLP-1 RAs weight loss drugs through thematic and CDA to understand how they shape public perceptions and behaviours. Our study found that the most prominent theme in the ads was the “HS” theme, with frequent references to medical credibility and efficacy. Our findings align with existing literature highlighting how direct-to-consumer pharmaceutical advertisements strategically leverage medical credibility and authoritative claims to enhance perceived legitimacy and consumer trust [32, 33]. Additionally, our study reflects recent insights into the growing role of digital health marketing and telehealth, which have rapidly expanded post-pandemic [34, 35]. These ads seek to reassure consumers about the safety and reliability of weight loss solutions by emphasizing FDA approval and clinical backing. This strategic emphasis on medical authority suggests that advertisers are leveraging the perceived infallibility of scientific validation to bolster consumer confidence.

Another significant finding was the emphasis on the “CS” theme. The frequent promotion of online consultations and telehealth services reflects a broader trend in healthcare delivery, particularly accelerated by the COVID-19 pandemic. According to Keesara et al. [34], the pandemic has drastically changed healthcare delivery models, increasing the adoption of telehealth services. This trend not only addresses accessibility and convenience but also positions these services as integral components of effective weight management. The integration of telehealth services signifies an adaptation to contemporary healthcare needs, ensuring that professional support is accessible to a wider audience regardless of geographic limitations.

The theme of “EA” highlights the efforts to make weight loss treatments appear straightforward and financially accessible. This strategy targets cost-conscious consumers by emphasizing special offers and discounts, creating a sense of urgency and making the treatments seem more financially feasible. Consumers are significantly influenced by price incentives in health-related purchases and it indicates that financial accessibility is a crucial factor in healthcare decisions [36].

The “EPI” theme taps into deeper psychological drivers such as confidence, transformation, and control. This approach is effective in motivating consumers but raises ethical concerns about exploiting vulnerabilities related to body image and self-esteem. The ethical critique underpinning our analysis emphasizes transparency and balanced communication in pharmaceutical advertising. While leveraging emotional appeals and authoritative endorsements may effectively persuade consumers, these strategies often omit balanced representations of potential risks and side effects, raising ethical concerns about consumer autonomy and informed decision-making [16]. Our analysis highlights this ethical tension, emphasizing the responsibility of pharmaceutical advertisers to provide truthful, comprehensive, and ethically sound communication.

Our findings resonate with established theories in advertising and social media marketing. As Bagozzi et al. [37] demonstrated, emotional triggers play a critical role in consumer decision-making, particularly for health-related products. This is evident in the GLP-1 RA advertisements analyzed in our study, which frequently evoke feelings of hope, transformation, and confidence to persuade potential consumers.

Moreover, Poels and Dewitte [38] emphasised that emotionally engaging advertisements enhance message recall and shape positive perceptions of the brand. The advertisements in this study effectively combine emotional language with visual imagery to reinforce desired outcomes, such as personal transformation and improved self-esteem. The importance of credibility is equally clear. Vrtana and Krizanova [33] argue that the use of authoritative endorsements and credible messaging builds trust among consumers, a strategy heavily employed in GLP-1 RA advertisements through the emphasis on FDA approval, medical consultations, and professional endorsements. These findings underscore how advertisers strategically leverage both emotional appeals and credibility-building tactics to shape public perceptions and influence health-related decisions.

Although this study did not directly compare brand-specific marketing strategies, we recognize that different GLP-1 RA brands may employ varied persuasive techniques to differentiate their products in the competitive weight-loss market. Future research could explore these distinctions in more detail.

Finally, future research should explore the long-term effects of advertising strategies on consumer behavior and public health. Studies should examine how balanced health communication, including potential risks and side effects, affects consumer trust and decision-making. Additionally, investigating consumer perspectives on weight loss treatments can provide insights into their effectiveness and ethical implications. Research should also assess how these ads impact different demographic groups, considering factors like age, gender, and socioeconomic status.

### Policy and practice implications

Our research offers unique insights with several important implications for policy and practice in regulatory oversight, public health education, telehealth integration, and ethical advertising. Table 5 summarizes the key focus areas and corresponding implications.

**Table 5:** Summary of key focus areas and corresponding policy and practice implications for social media advertisements of GLP-1 receptor agonist weight loss drugs

Primary focus	Key implications
Regulatory oversight	Enforce stricter guidelines for balanced advertisements, mandatory side effects disclosure, regular monitoring, and realistic expectations.
Transparency and accuracy	Provide comprehensive drug information including efficacy and risks, standardized side effects sections, and links to clinical trial data. Foster trust for long-term brand loyalty.
Public health education	Educate on integrating lifestyle changes with pharmaceuticals, promote holistic weight management, conduct workshops, create informational websites, and collaborate with healthcare providers.
Telehealth integration	Establish robust frameworks for telehealth evaluations, continuous monitoring, and immediate access to professionals. Ensure personalized care and continuous support.
Ethical advertising practices	Prioritize truthful, evidence-based information, avoid exaggerated claims, and be transparent about product limitations. Adhere to industry standards for responsible advertising.
Holistic approach to weight management	Emphasize psychological, social, and physical aspects of weight loss. Include mental health support and counseling in weight management programs.

Our study indicates that regulatory bodies like the FDA should enforce guidelines to ensure pharmaceutical ads provide a balanced view, including mandatory side effects disclosure and regular monitoring. This prevents misleading claims and ensures consumers are fully informed. Moreover, transparency and accuracy in advertisements are essential; ads should provide a comprehensive view of drug efficacy and risks. Standardized sections listing side effects and links to clinical trial data can help maintain consumer trust and promote long-term brand loyalty.

Additionally, public health campaigns should educate consumers on integrating lifestyle changes with pharmaceutical interventions, emphasizing a holistic weight management strategy. Workshops, informational websites, and collaborations with healthcare providers can disseminate accurate advice. The rising trend of telehealth should be supported with robust frameworks for evaluations, ongoing monitoring, and immediate access to professionals. This ensures personalized care and continuous support, which are crucial for effective weight management.

Furthermore, ethical advertising practices should be prioritized. Advertisers should provide clear, truthful information, avoid exaggerated claims, and be transparent about product limitations. Ethical practices can be reinforced through industry standards and certifications for responsible advertising.

Lastly, a holistic approach to weight management should be emphasized in both policy and practice. This means addressing psychological, social, and physical aspects of weight loss, including mental health support and counselling. For example, support groups, therapy sessions, and stress management programs can help individuals manage the psychological challenges associated with weight loss. We acknowledge that these policy recommendations are aspirational and may face significant challenges given the current regulatory environment, particularly in the United States, where oversight of direct-to-consumer advertising remains limited. Nevertheless, articulating these ideals is important to guide long-term advocacy efforts and inform future regulatory debates. Strengthening public awareness and fostering critical health literacy remain immediate actionable steps, even as systemic regulatory change evolves more gradually.

### Strengths and limitations

This study’s strengths include a comprehensive analysis combining qualitative and quantitative methods, providing a robust understanding of social media ad themes. Using real-world data from Facebook and Instagram ensures relevant findings. The interdisciplinary approach, incorporating thematic analysis, CDA, and quantitative analysis, offers a multifaceted perspective. However, the study is limited to ads from only two social media platforms, potentially missing strategies used in other media. Additionally, the manual coding process may introduce bias despite efforts to ensure rigor. Future studies should include more media types and consumer feedback for a more holistic understanding of these ads’ impact. This study focused specifically on Facebook and Instagram due to their high engagement rates and visibility in pharmaceutical marketing. However, we acknowledge that similar advertisements also appear on other platforms such as YouTube, TikTok, and commercial television, which may utilize different messaging strategies and reach different audiences. In addition, because direct-to-consumer pharmaceutical advertising is only permitted in a few countries (notably the United States and New Zealand), the majority of the ads we analyzed were likely targeted toward U.S. audiences. These geographic and platform-specific limitations should be considered when interpreting the findings.

## Conclusion

The CDA of social media ads for GLP-1 RAs weight loss drugs highlights the strategic use of medical authority and emotional appeals to highlight how marketing strategies construct narratives that may inform public perceptions and considerations related to weight management solutions. While these strategies effectively engage and persuade consumers, there is a crucial need for regulatory oversight and ethical standards to ensure balanced health communication and protect consumer interests. This research provides valuable insights for policymakers, healthcare providers, and pharmaceutical companies aiming to improve health communication strategies and promote responsible advertising practices. By addressing these areas, stakeholders can enhance public health outcomes and foster informed decision-making among consumers.

## Supplementary Information

Supplementary Material 1.

## Data Availability

Data will be made available on request.
