# Supplementary material for: Critical discourse analysis of social media advertisements for GLP-1 receptor agonist weight loss drugs: implications for public perceptions and health communication
Source: BMC Public Health. 2025 Sep 1;25:2996. doi: 10.1186/s12889-025-24197-8 (PMC12400570; doi:10.1186/s12889-025-24197-8)

Started running on 25 Aug 2023

Platforms

HRT Medics

Sponsored

Library ID: 317934367321005

Transform your weight and reclaim your health with the ultimate medical weight loss solution. For just $199/month unlock a new you!

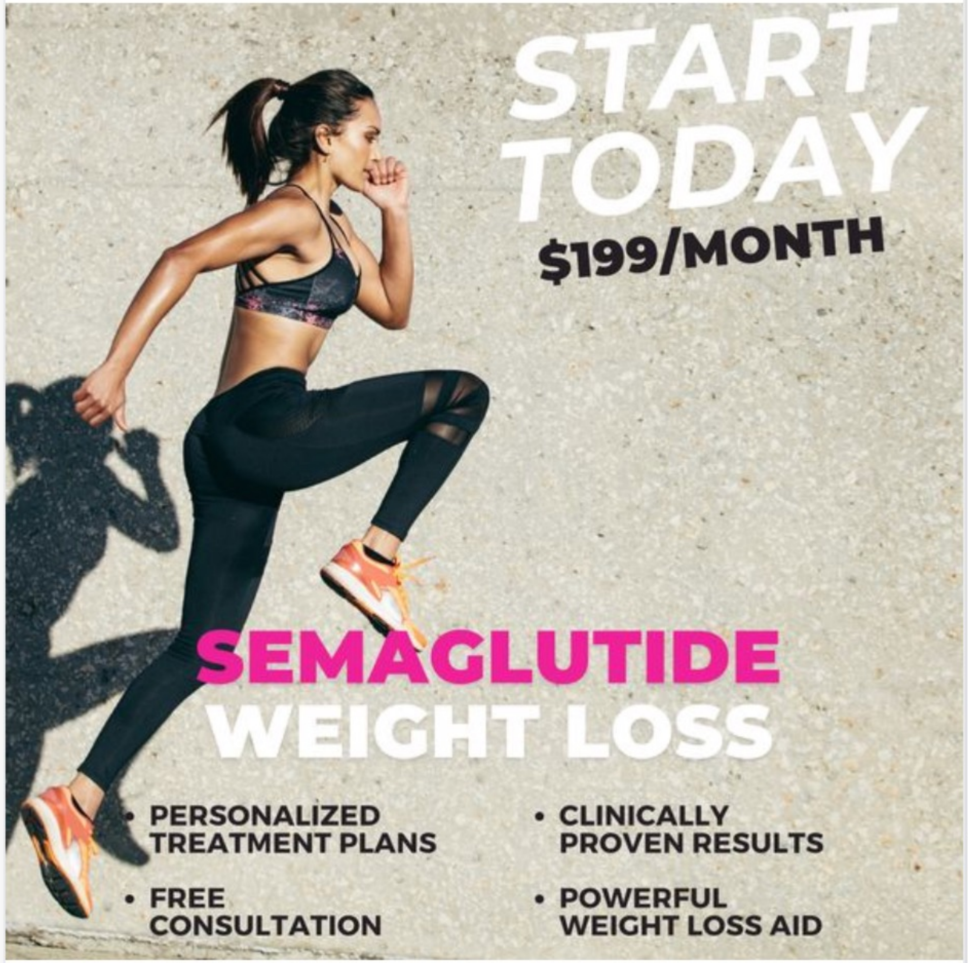

FB.ME

Reclaim Your Health: Join Our $199/month Weight

Sign up

Library ID: 758354839662274

Active

Started running on 10 Jan 2024

Platforms

Maximus

Sponsored

Library ID: 758354839662274

Use Tirzepatide to lose up to 22.5% of your body weight.

Prescription products require an online consultation with a doctor. Results may vary.

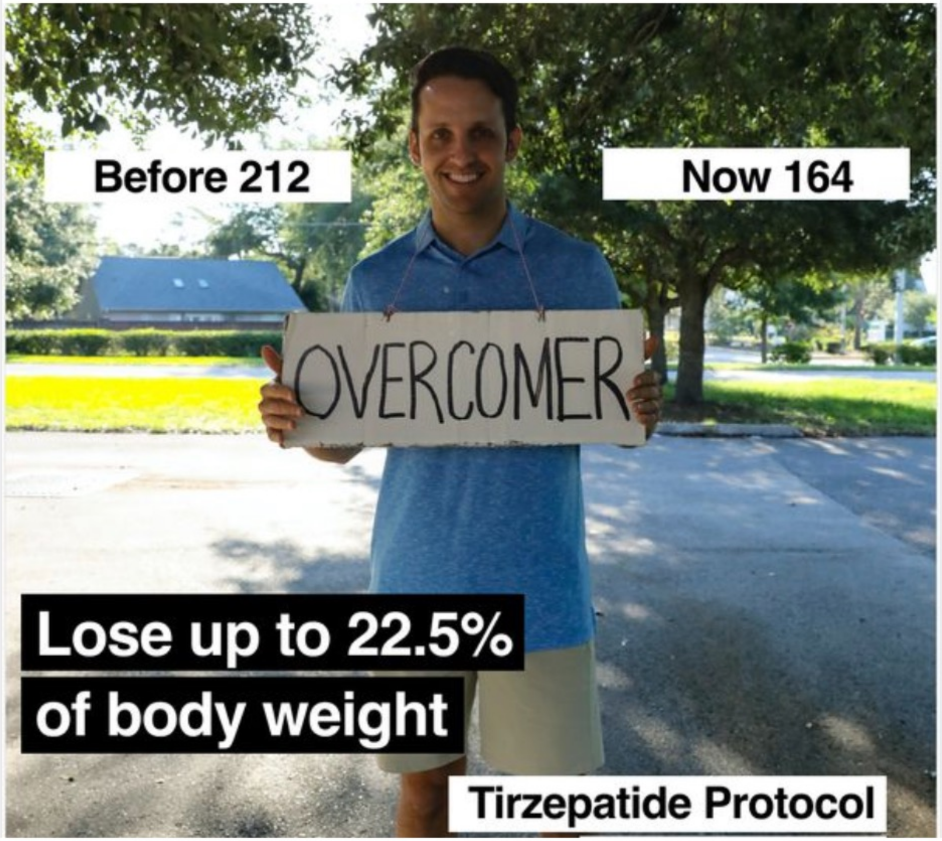

Ad details

I

dermal_aesthetics_supplies

Sponsored

Library ID: 785322789690763

•••

We got pen available in stock, order now and gain 7% discount, let's enjoy healthy life together and be happy and healthy.

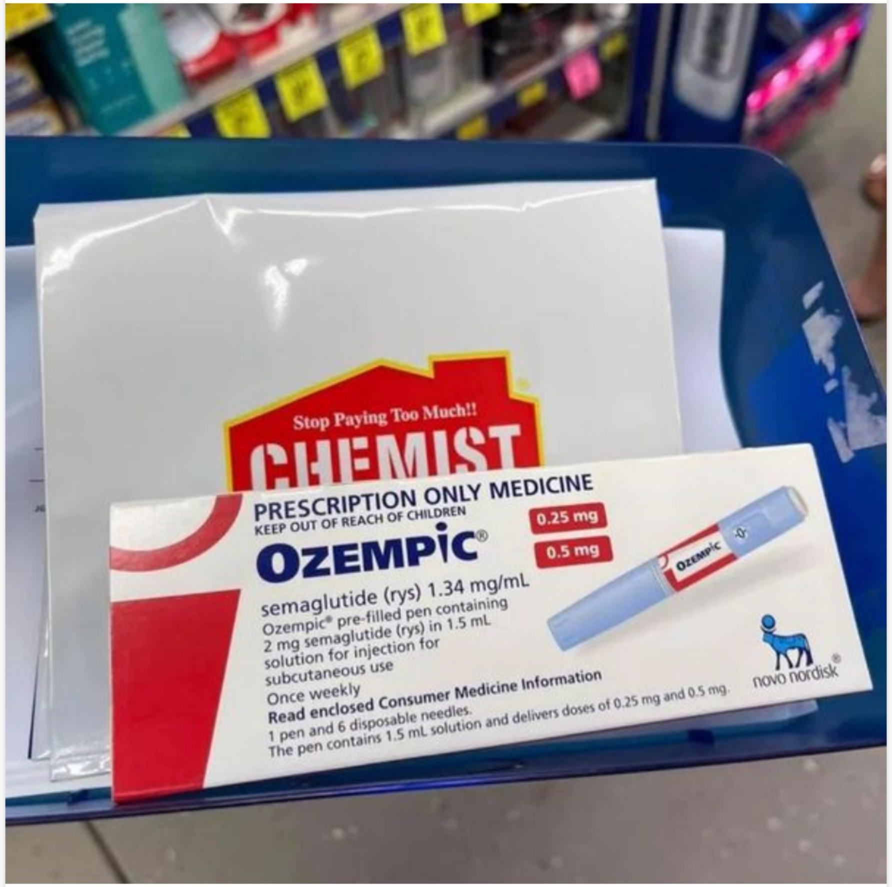

WHATSAPP

aesthetics_dermal_supplies9

Send Whats...

Library ID: 3699992303569074

Active

Started running on 22 Jan 2024

Platforms

This ad has multiple versions

Southern Ketamine and Wellness

Sponsored

Library ID: 3699992303569074

💫 Mounjaro is a breakthrough in weight loss medication. Experience significant weight reduction, fewer serious side effects, and a new level of confidence.

Join thousands who have found success with Mounjaro– start your transformation today! 🌼

...

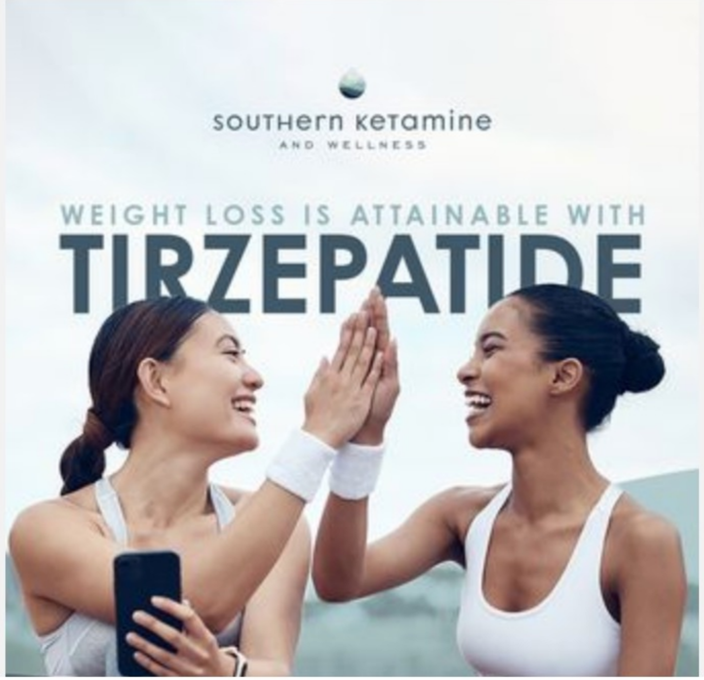

Supplement: Supplementary file 1 — Supplementary Material 1. [file 12889_2025_24197_MOESM1_ESM.pdf]
